# The “Gender Factor” in Wearing-Off among Patients with Parkinson's Disease: A Post Hoc Analysis of DEEP Study

**DOI:** 10.1155/2015/787451

**Published:** 2015-01-20

**Authors:** Delia Colombo, Giovanni Abbruzzese, Angelo Antonini, Paolo Barone, Gilberto Bellia, Flavia Franconi, Lucia Simoni, Mahmood Attar, Emanuela Zagni, Shalom Haggiag, Fabrizio Stocchi

**Affiliations:** ^1^Novartis Farma S.p.A., Origgio, Varese 21040, Italy; ^2^Department of Neurosciences, University of Genoa, Genoa 16132, Italy; ^3^Department of Parkinson's Disease, IRCCS San Camillo, Venice 30126, Italy; ^4^Scuola Medica Salernitana, Università degli Studi di Salerno, Salerno 84100, Italy; ^5^Department of Biomedical Science, University of Sassari, Sassari 07100, Italy; ^6^MediData srl, Modena 41123, Italy; ^7^Department of Neurology, San Camillo-Forlanini Hospital, Rome 00151, Italy; ^8^Department of Neurology, Institute of Research and Medical Care, IRCCS San Raffaele, Rome 00163, Italy

## Abstract

*Background*. The early detection of wearing-off in Parkinson disease (DEEP) observational study demonstrated that women with Parkinson's disease (PD) carry an increased risk (80.1%) for wearing-off (WO). This post hoc analysis of DEEP study evaluates gender differences on WO and associated phenomena. *Methods*. Patients on dopaminergic treatment for ≥1 year were included in this multicenter observational cross-sectional study. In a single visit, WO was diagnosed based on neurologist assessment as well as the use of the 19-item wearing-off questionnaire (WOQ-19); WO was defined for scores ≥2. Post hoc analyses were conducted to investigate gender difference for demographic and clinical features with respect to WO. *Results*. Of 617 patients enrolled, 236 were women and 381 were men. Prevalence of WO was higher among women, according to both neurologists' judgment (61.9% versus 53.8%, *P* = 0.045) and the WOQ-19 analysis (72.5% versus 64.0%, *P* = 0.034). In patients with WO (WOQ-19), women experienced ≥1 motor symptom in 72.5% versus 64.0% in men and ≥1 nonmotor symptom in 44.5% versus 36.7%, in men. * Conclusions*. Our results suggest WO as more common among women, for both motor and nonmotor symptoms. Prospective studies are warranted to investigate this potential gender-effect.

## 1. Background

Parkinson's disease (PD) is one of the most common age-related progressive neurodegenerative disorders, with no identifiable cause. PD is slightly more common in men than in women in most studies, usually ranging from a 1.2 : 1 ratio up to a 1.5 : 1 ratio [1], and men seem to be at higher risk for PD [2–4]. The reasons for the increased risk in men are not known; probably “male lifestyle” could account for some of the excess incidence in men [5]. Alternatively, there is increasing evidence from in vitro as well as clinical studies in humans that estrogen may be neuroprotective [6]. Sex-related differences have been reported in the onset of symptoms and type of motor symptoms as well as in medication use [7, 8]. Notably, normal human basal ganglia, specifically within the dopamine system, are sexually dimorphic and that may influence the onset and progression of PD [8].

Levodopa is the most efficacious treatment in the management of PD. Unfortunately, chronic use of traditional levodopa/dopa decarboxylase inhibitor formulations is associated with the development of motor complications, such as wearing-off (WO) and dyskinesia that occur in the majority of PD patients. The WO effect, or end-of-dose failure, refers to a decrease in the length of time that each dose of levodopa controls symptoms. “Off” states that result in motor and nonmotor symptoms, freezing of gait (FOG), and falling are disabling for many patients. Considered to be the major source of disability in PD patients, recognition of these complications is critical in order to develop different strategies designed not only to treat these problems when they develop, but also to prevent troublesome complications associated with potential risk factors.

We previously conducted an observational, cross-sectional, multicenter study called* Early DEtection of wEaring off in Parkinson disease* (DEEP Study) [9, 10]. The primary goal was to look at the frequency of WO phenomena among a wide population of Italian patients with PD and secondly to assess associated phenomena, such as FOG and nonmotor symptoms, as well as assessing the impact on patient's QoL. In our sample WO occurred since the early years of the disease; furthermore younger age, unified Parkinson's disease rating scale (UPDRS) part II score, duration of anti-Parkinson (APD) treatment, and female gender were found significantly associated with WO. Our data showed women having an 80.1% higher risk of experiencing WO than men [9]. This exploratory study aimed to further examine what role the “gender factor” in WO would play, that is, to characterize disparities between women and men in frequency and features of WO symptoms. For this purpose, we conducted a post hoc analysis of the DEEP study database. This study is also part of the gender-medicine project (METAGEM), being carried out with the aim to describe clinical outcomes and therapeutic approach by gender, through the analysis of observational studies conducted in Italy, among different therapeutic areas [11].

## 2. Methods

Patients prospectively recruited were men and women aged 18 years or older, with PD (Hoehn and Yahr Stages 1–5), nondemented, under levodopa (LD), and/or dopamine agonists (DAs) therapy for ≥1 year before the study screening. The full DEEP study design, including complete inclusion/exclusion criteria, has been published [9]. The study was conducted in accordance with Good Clinical Practice and the Declaration of Helsinki. The study protocol and amendments were approved by each local Ethics Committees or Institutional Review Boards of all 37 participating centers (both academic and hospital based) across Italy (Additional file 1). All patients provided written, informed consent before study participation.

During a single visit, neurologists experienced in movement disorders and previously subjected to targeted training acquired standard demographic and detailed clinical information on all participants who met the criteria, via a structured interview and by examination. Therapy was expressed in terms of Levodopa equivalent daily dose (LEDD) [12]. Subjects were assessed with the UPDRS and the Hoehn and Yahr scale (H&Y). The diagnosis of WO was made on both the neurologist evaluation and the basis of the patient self-assessment using the Italian version of the* 19-item Wearing-Off Questionnaire* (WOQ-19) [10]. The WOQ-19 consists of 9 items assessing fluctuations of motor symptoms, including tremor, difficulty in speech, weakness, problems with balance, slowness, reduced dexterity, general stiffness, muscle cramps, and difficulty getting out of the chair and 10 items assessing fluctuations of nonmotor symptoms, including anxiety, sweating, mood changes, numbness, panic attacks, cloudy mind/dullness of thinking, abdominal discomfort, experience hot and cold, pain, and aching [10]. For each item, patients were asked to tick whether symptoms were present and whether they improved after the following dose of anti-Parkinson treatment: a cutoff of ≥2 improved symptoms has been previously established to make diagnosis of WO [10].

## 3. Statistical Analysis

Descriptive analyses were mean and standard deviation (SD) or median and interquartile range (IQR). Only the fully completed scales were considered evaluable for the statistical analysis. Gender differences analyses were performed on all patients who responded to inclusion-exclusion criteria. Differences in demographics/baseline characteristics between patients with WO and patients with no WO (as assessed using WOQ-19 and the neurologist assessment) were estimated using *t*-test for continuous data and the Chi-square test for categorical data. As post hoc analyses, all* P* values presented are exploratory. Patients with missing data in selected parameters were not evaluated for those parameters. All analyses were performed with SAS v. 9.2 and Enterprise Guide 4.3.

## 4. Results

Of the 634 patients screened, 617 (97.3%) met the inclusion criteria: 236 (38.2%) were women and 381 (61.8%) were men (Table 1). The excluded patients did not differ significantly on demographic or clinical parameters with respect to the study population [9]. Baseline demographic data are provided in Tables 1 and 2. Evaluation of gender differences in the study sample indicated no difference in age, history of concomitant diseases, caregiver support, body mass index, or coffee consumption. As shown in Table 1, women were less likely to be married/cohabiting (*P* = 0.0001) and have less education (*P* = 0.0002). In almost the third of cases women were housewives and were less likely to be employed (*P* = 0.0046). Men were more likely to be past (*P* < 0.0001) or current smokers (*P* = 0.0059) and to be frequent alcohol consumers (*P* < 0.0001), although more often engaged in physical activity on a regular basis (*P* = 0.0005).

A shown in Table 2, disease duration, H&Y staging, and UPDRS and MMSE scores were statistically indistinguishable between both groups. Postural instability/gait difficulties (PIGD) phenotype was found in 53.0% of the whole sample, although more prevalent in women (57.0% versus 50.5%, *P* = 0.005), while the tremor dominant (TD) phenotype was more frequently attributed to men (29.0% versus 41.4%, *P* = 0.005).

**Figure 1:**
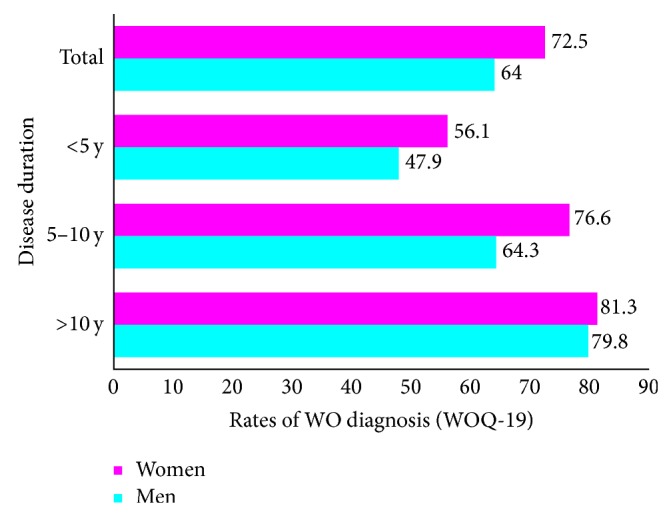
Rates of WO diagnosis according to WOQ-19 stratified by disease duration: women versus men.

**Figure 2:**
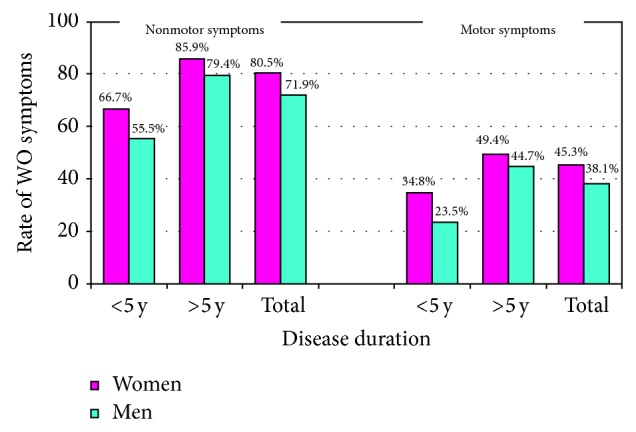
Rates of motor and nonmotor WO symptoms: women versus men.

Prevalence of WO was higher among women, according to both neurologists' judgment (61.9% versus 53.8%, *P* = 0.0495) and WOQ-19 analysis (72.5% versus 64.0%, *P* = 0.034) (Figure 1). Taking into account the symptoms reported as usually improving after the following dose of anti-Parkinson agents (APD), otherwise defined as WO symptoms, 3.3 ± 2.5 versus 3.0 ± 2.6 were motor symptoms (*P* = 0.222) and 1.2 ± 1.8 versus 0.9 ± 1.5 were nonmotor symptoms (*P* = 0.0125), respectively, in women and men. The frequency of symptoms reported by patients through the WOQ-19, stratified by gender and disease duration, is listed in Table 3. Among patients with a diagnosis of WO according to WOQ-19, women experienced ≥1 motor symptom in 72.5% versus 64.0% in men and ≥1 nonmotor symptom in 44.5% versus 36.7%, respectively, in men (Figure 2).

With regard to APD therapy, no differences were observed in the use of the different classes of APDs (*P* = 0.4457) or for LEDD values (844.2 ± 679.0 versus 874.4 ± 749.1, *P* = 0.622).

Finally, women reported significantly higher PDQ-8 scores than men (31.3 ± 18.4 versus 27.7 ± 19.1, *P* = 0.023).

## 5. Discussion

WO is an important feature of PD, often marking the end of the “honeymoon period.” The WO manifestations can be extremely heterogeneous from subject to subject, and early recognition allows timely optimization of treatment that may impact patient care and long-term clinical outcomes. The reasons underlying these complications are not fully understood and well-recognized risk factors for the development of WO and dyskinesias include young age at onset, low body weight, severity of disease higher levodopa dose, association of levodopa with entacapone, once daily intake of levodopa, duration of levodopa therapy, more severe UPDRS Part II, and female gender [9, 13–15]. This paper was dedicated to assess gender differences in WO and is based on the post hoc analysis of the DEEP study, a large epidemiological survey that enrolled more than 600 patients with PD across Italy. This study specifically investigated for WO and shows that women are more likely to suffer from WO symptoms as compared to men and that female gender confers an increased risk for WO equal to 80.1% [9]. This finding was confirmed both by the neurologist assessment (61.9% versus 53.8%, *P* = 0.049) and by patients themselves thorough the WOQ-19 (72.5% versus 64.0%, *P* = 0.034). Among patients with WO (WOQ-19), women more frequently complained of ≥1 motor (72.5% versus 64.0%) and ≥1 nonmotor (44.5% versus 36.7%) symptoms of WO. Our findings have been supported in a previous clinic-based sample of patients with PD, where WO symptoms have been shown to occur more frequently in women (46% versus 29%, *P* = 0.02) [16]. Furthermore, durations to develop WO and dyskinesia were shown to be shorter in women compared to men, as well as disease progression being slightly faster for women [17]. A similar “gender effect” has already been shown for the occurrence of levodopa-induced dyskinesias, as it has been claimed that dyskinesias occur more frequently in women if disease duration is >5 years [18, 19] and with a shorter time latency than men [20]. Otherwise, in a large British community-based study, men and women did not differ in the occurrence of motor fluctuations or dyskinesias [13]. In a recent post hoc analysis of a large prospective trial, female gender was identified as a specific predicting factor for dyskinesias and WO as well, and the authors hypothesized that it would reflect increased levodopa concentrations in women because of lower body weight [15]. This interesting hypothesis, in addition to providing an explanation of the association with female gender, would place the emphasis on a parameter, such as body weight, which in clinical practice is generally overlooked for therapy decisions. Data available from our observational study do not allow analyzing this aspect, as patients were undergoing very heterogeneous treatments and were poorly comparable to one another.

In DEEP population women and men were substantially homogeneous for age, disease duration, H&Y staging, and neurological disability (UPDRS; MMSE); otherwise smoking habits, alcohol consumption, and physical activity differed, and this reflects what is observed in the general population [21]. To our knowledge no data indicate a direct association of these social factors with WO; nevertheless, it is plausible that they might influence PD treatment and vice versa, and we cannot exclude possible association with the development of WO and contribution for the differences between women and men. Large cohort studies would address these hypotheses.

DEEP women reported poorer QoL than men (*P* = 0.023), probably due to WO, as we have previously reported that the number of motor symptoms (0.34008; *P* < 0.0001) and nonmotor symptoms of WO (0.33595; *P* < 0.0001) correlates with PDQ-8 score and that by linear regression analysis the presence of each additional WO symptom, as identified by WOQ-19, corresponds to an increase of 1.15 points of the PDQ-8 score (*P* < 0.0001) [9].

According to treatment guidelines in PD, the symptomatic control of WO should be obtained by increasing and fractionating the dose of levodopa or by the addition of other drugs, such as DAs, catechol-O-methyl transferase (COMT) inhibitors, or monoamine oxidase inhibitors [22–24]. Surprisingly, in front of the higher prevalence of WO, DEEP women were indistinctly treated as men, both in terms of classes of APD and by LEDD analysis. This could have several explanations. First, women and men may exhibit different pharmacological response to APD, making a direct comparison between women and men difficult. Although gender differences are generally not acknowledged in published clinical guidelines of PD management, evidence of gender disparities in medication response, pharmacokinetics, and tolerability suggests the need for paying more attention to differences between women and men in clinical management. Levodopa treatment has been found to significantly improve motor function in women more than in men [25]. Studies suggest that women have greater levodopa bioavailability [26, 27] and different clearance of dopaminergic agents, which may play a role in gender-specific dosing of PD medications and may explain why women are more likely to have levodopa-related dyskinesias [18, 19, 28]. The COMT inhibitors have also been reported to have different tolerability profiles based on gender, which may result from disparities in optimal levodopa dosage [29, 30].

Second, the higher frequency of WO among women may reflect a state of undertreatment. If this is the case, we may interpret it as a more “gentle” treatment approach adopted for women with PD, compared to men, perhaps due to safety and tolerability concerns. This hypothesis is in line with the findings of a large population-based study including more than 25,000 patients with PD (Thomson Reuter's Marketscan), as men were found to be more likely to be treated with an APD in the first year after diagnosis (72.9% versus 67.5%, *P* < 0.0001) and to be treated adjunctively with APD (23.9% versus 21.3%, *P* < 0.0001), compared to women [31]. Third, since nonmotor symptoms appeared to more heavily affect WO phenomena in women, clinicians could be oriented to prescribe other nondopaminergic therapies, rather than APD. Among DEEP women, the analysis of individual WO symptoms shows the greater prevalence of nonmotor symptoms such as “anxiety” (23.7% versus 15.2%), “mood changes” (22.0% versus 15.5%), and “pain” (15.7% versus 7.3%). This supports previous observations, where women were found more likely than man to be prescribed an antidepressant (53.1% versus 38.5%, *P* < 0.0001), an antipsychotic (19.2% versus 14.4%, *P* < 0.0001), and an anxiolytic (19.6% versus 14.7%, *P* < 0.0001) [31].

Given the complexity of PD, as well as the potential for patient characteristics to affect WO symptoms and their management, our study allows an initial assessment of the “gender effect” in the WO manifestations and management in the real-world setting. Nevertheless, our study has a number of limitations: (a) as a post hoc analysis it was not originally designed to assess gender differences of WO symptoms; (b) statistical analysis was mainly descriptive and *P* values are only explorative; (c) there are some limitations of the original study, such as patients selection bias and no analysis of non-APD medications; furthermore only symptoms included in WOQ-19 were assessed, which precludes making assumptions about the contribution of other symptoms.

In conclusion, sex differences in WO among subjects with PD appear to exist and may have implications for the optimal utilization of APD therapy. Our results further put in perspective current clinical management of WO symptoms, raising concern about the appropriateness of treatment approach and also the risk of generalizing data derived from trials in which women are often underrepresented. Further research into the long-term implications of these disparities is needed, and studies such as ours, although limited, suggest that “gender effect” should be carefully considered in designing clinical studies in PD.

## Figures and Tables

**Table 1:** Demographic and lifestyle data of DEEP study population: women versus men.

	Women *N* = 236	Men *N* = 381	*P*
Age, mean ± SD	67.0 ± 8.9	66.6 ± 9.4	0.6602
Marital status, *N* (%)^#^			
Married/cohabiting	168 (71.2)	321 (84.3)	0.0001
Single	16 (6.8)	27 (7.1)	
Divorced/separated	5 (2.1)	13 (3.4)	
Widowed	46 (19.5)	15 (3.9)	
Employment status, *N* (%)			
Employed	23 (9.7)	69 (18.1)	0.0046
Unemployed	1 (0.4)	6 (1.6)	
Retired	130 (55.1)	288 (75.6)	
Housewife	75 (31.8)	—	
Other	7 (3.0)	18 (4.7)	
Education, *N* (%)			0.0002
≤5 years	109 (46.2)	113 (29.7)	
6–8 years	54 (22.9)	122 (32.0)	0.0002
>8 years	73 (30.9)	146 (38.3)	0.0008
Caregiver assistance, *N* (%)	49 (20.8)	63 (16.5)	0.2050
Smoking habit, *N* (%)^#^			<0.0001
Smokers	15 (6.4)	37 (9.7)	0.0059
Ex-smokers	32 (13.6)	149 (39.1)	<0.0001
Nonsmokers	183 (77.5)	189 (49.6)	
Coffee consumers, *N* (%)	162 (68.6)	264 (69.3)	0.6622
Alcohol consumers, *N* (%)	84 (35.6)	240 (63.0)	<0.0001
Regular physical activity, *N* (%)	55 (23.3)	140 (36.7)	0.0005
BMI, mean ± SD	24.9 ± 4.5	26.4 ± 3.5	
Concomitant diseases, *N* (%)	145 (61.4)	205 (53.8)	0.0764
Hypertension	75 (31.8)	109 (28.6)	
Ischemic heart Disease	91 (38.6)	137 (36.0)	
Diabetes mellitus	20 (8.5)	34 (8.9)	
Neoplasms	7 (3.0)	13 (3.4)	
Cerebrovascular disease	5 (2.1)	4 (1.0)	
Obesity	3 (1.3)	7 (1.8)	
Psychiatric disorders	9 (3.8)	9 (2.4)	
Other	57 (24.2)	72 (18.9)	

^#^Missing data for <10 patients/variable.

**Table 2:** Gender differences on clinical aspects of DEEP population: women versus men.

	Women *N* = 236	Men *N* = 381	*P*
Disease duration, mean ± SD			0.389
<5 years, *N* (%)	66 (28.0)	119 (31.2)	
>5 years, *N* (%)	170 (72.0)	262 (68.8)	
H&Y, *N* (%)^#^			0.291
≤2	134 (56.8)	233 (61.1)	
≥2.5	101 (42.8)	148 (38.6)	
UPDRS total score, mean ± SD	36.2 ± 17.7	38.1 ± 17.4	0.223
Part I	2.3 ± 2.0	2.1 ± 1.9	0.287
Part II	11.1 ± 6.4	11.3 ± 6.0	0.621
Part III	23.0 ± 11.6	24.6 ± 11.8	0.129
PD subtypes, *N* (%)^§^			0.006
TD	62 (26.3)	142 (37.3)	
PIGD	120 (50.8)	172 (45.1)	0.0149
IND	29 (12.3)	28 (7.3)	0.0041
MMSE, mean ± SD	28.1 ± 1.67	28.2 ± 1.62	0.306
Anti-Parkinson drugs classes, *N* (%)			0.4457
LD monotherapy	29 (12.3)	35 (9.2)	
DA monotherapy	11 (4.7)	15 (3.9)	
LD + DA	152 (64.4)	234 (61.4)	
MAOIs	84 (35.6)	157 (41.2)	
COMT inhibitors	79 (33.5)	132 (34.6)	
LEDD, mg mean ± SD	844.2 ± 679.0	874.4 ± 749.1	0.622
Diagnosis of WO by neurologists, *N* (%)	146 (61.9)	205 (53.8)	0.0495
<5 years disease duration	21 (31.8)	38 (31.9)	0.987
>5 years disease duration	125 (73.5)	167 (63.7)	0.034
Diagnosis of WO by WOQ-19, *N* (%)	171 (72.5)	244 (64.0)	0.034
<5 years disease duration	37 (56.1)	57 (47.9)	0.2875
>5 years disease duration	134 (78.8)	187 (71.4)	0.0834
PDQ-8, mean ± SD	31.3 ± 18.4	27.7 ± 19.1	0.023

SD: standard deviation; H&Y: Hoehn and Yahr staging; UPDRS: unified Parkinson's disease rating scale; PD: Parkinson's disease; TD: tremor dominant; PIGD: postural instability and gait difficulties; IND: intermediate; MMSE: mini-mental state examination; LD: levodopa; DA: dopamine-agonist; MAOIs: monoamine oxidase B inhibitors; COMT: catechol-O-methyltransferase; LEDD: levodopa equivalent daily dose; WO: wearing-off; WOQ-19: 19-item wearing-off questionnaire; PDQ-8: 8-item Parkinson's disease questionnaire.

^§^PD subtypes could not be calculated for 39 males and 25 females. Paired post hoc Chi-squares take TD as reference.

^#^Missing data for <10 patients/variable.

**Table 3:** Frequency of experienced symptoms and WO symptoms, as reported by DEEP population, through the WOQ-19, stratified by disease duration: women versus men.

Disease duration	<5 years		>5 years		Total	
	(*N* = 185)		(*N* = 432)		(*N* = 617)	
	Women (*N* = 66)	Men (*N* = 119)	Women (*N* = 170)	Men (*N* = 262)	Women (*N* = 236)	Men (*N* = 381)
Referred motor symptoms by WOQ-19, mean ± SD						
Experienced	4.6 ± 2.2	4.8 ± 2.1	6.0 ± 1.9	5.9 ± 2.0	5.6 ± 2.1	5.5 ± 2.1
WO	2.0 ± 2.1	2.0 ± 2.4	3.8 ± 2.4	3.5 ± 2.6	3.3 ± 2.5	3.0 ± 2.6
Referred nonmotor symptoms by WOQ-19, mean ± SD						
Experienced	2.9 ± 2.0	2.3 ± 2.2	4.0 ± 2.3	3.0 ± 2.2	3.7 ± 2.3	2.8 ± 2.2
WO	0.7 ± 1.3	0.5 ± 1.1	1.4 ± 1.9	1.1 ± 1.6	1.2 ± 1.8	0.9 ± 1.5
Experienced and WO symptoms, mean ± SD						
Experienced	7.5 ± 3.6	7.1 ± 3.8	9.9 ± 3.6	8.9 ± 3.7	9.3 ± 3.8	8.3 ± 3.8
WO	2.7 ± 2.8	2.4 ± 3.1	5.2 ± 3.9	4.5 ± 3.8	4.5 ± 3.8	3.9 ± 3.7
Experienced and WO symptoms, *n* (%)						
Tremor						
Experienced	48 (72.7)	81 (68.1)	117 (68.8)	180 (68.7)	165 (69.9)	261 (68.5)
WO	29 (43.9)	40 (33.6)	96 (56.5)	127 (48.5)	125 (53.0)	167 (43.8)
Difficulty in speech						
Experienced	14 (21.2)	51 (42.9)	62 (36.5)	152 (58.0)	76 (32.2)	203 (53.3)
WO	8 (12.1)	21 (17.6)	28 (16.5)	76 (29.0)	36 (15.3)	97 (25.5)
Weakness						
Experienced	48 (72.7)	72 (60.5)	129 (75.9)	184 (70.2)	177 (75.0)	256 (67.2)
WO	14 (21.2)	27 (22.7)	68 (40.0)	102 (38.9)	82 (34.7)	129 (33.9)
Problems with balance						
Experienced	24 (36.4)	49 (41.2)	110 (64.7)	136 (51.9)	134 (56.8)	185 (48.6)
WO	7 (10.6)	18 (15.1)	48 (28.2)	64 (24.4)	55 (23.3)	82 (21.5)
Slowness of movements						
Experienced	50 (75.8)	90 (75.6)	157 (92.4)	227 (86.6)	207 (87.7)	317 (83.2)
WO	27 (40.9)	42 (35.3)	122 (71.8)	153 (58.4)	149 (63.1)	195 (51.2)
Reduced dexterity						
Experienced	36 (54.5)	85 (71.4)	143 (84.1)	219 (83.6)	179 (75.8)	317 (79.8)
WO	18 (27.3)	37 (31.1)	101 (59.4)	145 (55.3)	119 (50.4)	182 (47.8)
General stiffness						
Experienced	22 (33.3)	49 (41.2)	98 (57.6)	168 (64.1)	120 (50.8)	217 (57.0)
WO	12 (18.2)	27 (22.7)	80 (47.1)	122 (46.6)	92 (39.0)	149 (39.1)
Muscle cramps						
Experienced	36 (54.5)	57 (47.9)	100 (58.8)	138 (52.7)	136 (57.6)	195 (51.2)
WO	11 (16.7)	11 (9.2)	37 (21.8)	48 (18.3)	48 (20.3)	59 (15.5)
Difficulty getting out of chair						
Experienced	24 (36.4)	36 (30.2)	98 (57.6)	137 (52.3)	122 (51.7)	173 (45.4)
WO	6 (9.1)	11 (9.2)	62 (36.5)	79 (30.1)	68 (28.8)	90 (23.6)
Anxiety						
Experienced	42 (63.6)	49 (41.2)	112 (65.9)	123 (46.9)	154 (65.3)	172 (45.1)
WO	10 (15.1)	13 (10.9)	46 (27.1)	45 (17.2)	56 (23.7)	58 (15.2)
Sweating						
Experienced	16 (24.2)	30 (25.2)	85 (50.0)	102 (38.9)	101 (42.8)	132 (34.6)
WO	3 (4.5)	4 (3.4)	19 (11.2)	28 (10.7)	22 (9.3)	32 (8.4)
Mood changes						
Experienced	28 (42.4)	47 (39.5)	94 (55.3)	112 (42.7)	122 (51.7)	159 (41.7)
WO	8 (12.1)	12 (10.1)	44 (25.9)	47 (17.9)	52 (22.0)	59 (15.5)
Numbness						
Experienced	11 (16.7)	19 (16.0)	53 (31.2)	52 (19.8)	64 (27.1)	71 (18.6)
WO	2 (3.0)	2 (1.7)	16 (9.4)	17 (6.5)	18 (7.6)	19 (5.0)
Panic attacks						
Experienced	5 (7.6)	7 (5.9)	29 (17.1)	32 (12.2)	34 (14.4)	39 (10.2)
WO	1 (1.5)	1 (0.8)	11 (6.5)	9 (3.4)	12 (5.1)	10 (2.6)
Cloudy mind						
Experienced	17 (25.7)	22 (18.5)	59 (34.7)	96 (36.6)	76 (32.2)	118 (31.0)
WO	4 (6.1)	3 (2.5)	21 (12.3)	41 (15.6)	25 (10.6)	44 (11.5)
Abdominal discomfort						
Experienced	19 (28.8)	30 (25.2)	53 (31.2)	48 (18.3)	72 (30.5)	78 (20.5)
WO	1 (1.5)	2 (1.7)	11 (6.5)	13 (5.0)	12 (5.1)	15 (3.9)
Feelings of hot/cold						
Experienced	11 (16.7)	16 (13.4)	51 (30.0)	53 (20.2)	62 (26.3)	69 (18.1)
WO	2 (3.0)	2 (1.7)	14 (8.2)	12 (4.6)	16 (6.8)	14 (3.7)
Pain						
Experienced	29 (43.9)	26 (21.8)	73 (42.9)	75 (28.6)	102 (43.2)	101 (26.5)
WO	8 (12.1)	5 (4.2)	29 (17.1)	23 (8.8)	37 (15.7)	28 (7.3)
Aching						
Experienced	14 (21.2)	31 (26.1)	67 (39.4)	88 (33.6)	81 (34.3)	119 (31.2)
WO	5 (7.6)	11 (9.2)	33 (19.4)	41 (15.6)	38 (16.1)	52 (13.6)

**Table 4:** Members of the DEEP study group.

First name	Last name	Affiliation	City
Fabrizio	Stocchi	Dipartimento di Neurologia IRCCS San Raffaele Pisana di Roma	Roma
Laura	Vacca	Dipartimento di Neurologia IRCCS San Raffaele Pisana di Roma	Roma
Peter P.	Pramstaller	Neurologia Ospedale di Bolzano	Bolzano
Maurizio	Facheris	Neurologia Ospedale di Bolzano	Bolzano
Mario	Guidotti	Neurologia Ospedale Valduce	Como
Elisabetta	Corengia	Neurologia Ospedale Valduce	Como
Giulio	Riboldazzi	Neurologia Ospedale di Circolo e Fondazione Macchi	Varese
Serena	Leva	Neurologia Ospedale di Circolo e Fondazione Macchi	Varese
Alberto	Priori	Neurologia Fondazione Ospedale Maggiore Policlinico	Milano
Filippo	Cogiamanian	Neurologia Fondazione Ospedale Maggiore Policlinico	Milano
Gianni	Pezzoli	Centro Parkinson Istituti Clinici di Perfezionamento	Milano
Canesi	Margherita	Centro Parkinson Istituti Clinici di Perfezionamento	Milano
Alberto	Albanese	Neurologia I Fondazione IRCCS Istituto Nazionale Neurologico C. Besta; Università Cattolica del Sacro Cuore	Milano
Paola	Soliveri	Neurologia I Fondazione IRCCS Istituto Nazionale Neurologico C. Besta	Milano
Daniele	Picco	Neurologia Riabilitativa Fondazione Salvatore Maugeri IRCCS Veruno	Veruno
Fabrizio	Pisano	Neurologia Riabilitativa Fondazione Salvatore Maugeri IRCCS Veruno	Veruno
Leonardo	Scarzella	Neurologia Ospedale Evangelico Valdese	Torino
Alessia	Tavella	Neurologia Ospedale Evangelico Valdese	Torino
Leonardo	Lopiano	Neurologia 4 A.S.O. Molinette	Torino
Maurizio	Zibetti	Neurologia 4 A.S.O. Molinette	Torino
Michele	Tinazzi	U.O. Neurologia Ospedale Civile Maggiore-Borgo Trento	Verona
Sarah	Ottaviani	U.O. Neurologia Ospedale Civile Maggiore-Borgo Trento	Verona
Franco	Valzanìa	Clinica Neurologica Nuovo Ospedale Sant'Agostino-Estense	Modena
Sara	Contardi	Clinica Neurologica Nuovo Ospedale Sant'Agostino-Estense	Modena
Rocco	Quatrale	UO Neurologia Arcispedale Sant'Anna	Ferrara
Mariachiara	Sensi	UO Neurologia Arcispedale Sant'Anna	Ferrara
Roberto	Ceravolo	U.O. Neurologia Ospedale Santa Chiara	Pisa
Carlo	Rossi	U.O. Neurologia Ospedale Santa Chiara	Pisa
Massimo	Cincotta	Neurologia Azienda Sanitaria Firenze-S Giovanni di Dio (SGDD)	Firenze
Paola	Vanni	Neurologia Azienda Sanitaria Firenze-S Giovanni di Dio (SGDD)	Firenze
Ubaldo	Bonuccelli	U.O. Neurologia Ospedale Versilia	Camaiore
Paolo	Del Dotto	U.O. Neurologia Ospedale Versilia	Camaiore
Maria Gabriella	Ceravolo	Clinica Neuroriabilitazione Az. Ospedali Riuniti	Ancona
Marianna	Capecci	Clinica Neuroriabilitazione Az. Ospedali Riuniti	Ancona
Roberta	Marchese	Centro Parkinson-Dipartimento Neuroscienze Università degli Studi di Genova	Genova
Tiziano	Tamburini	Centro Parkinson-Dipartimento Neuroscienze Università degli Studi di Genova	Genova
Astrid	Thomas	CeSI-Centro Studi Invecchiamento Fondazione Università Gabriele D'Annunzio	Chieti
Iole	Borrelli	CeSI-Centro Studi Invecchiamento Fondazione Università Gabriele D'Annunzio	Chieti
Roberto	Marconi	Neurologia Ospedale della Misericordia	Grosseto
Simone	Gallerini	Neurologia Ospedale della Misericordia	Grosseto
Paolo	Stanzione	Clinica Neurologica Università di Roma Tor Vergata–IRCCS S Lucia	Roma
Valerio	Pisani	Clinica Neurologica Policlinico Tor Vergata	Roma
Anna Rita	Bentivoglio	Neurologia Università Cattolica S. Cuore Policlinico Gemelli	Roma
Giovanna	Lorìa	Neurologia Università Cattolica S. Cuore Policlinico Gemelli	Roma
Maria Francesca	De Pandis	U.O. Riabilitazione Parkinson Ospedale San Raffaele Cassino	Cassino
Giovanna	Federici	U.O. Riabilitazione Parkinson Ospedale San Raffaele Cassino	Cassino
Valentino	Manzo	Neurologia, Padiglione F,Amb UVA AORN A. Cardarelli	Napoli
Alfonso	Mauro	Struttura Semplice Malattia di Parkinson AORN San Giovanni di Dio e Ruggi d'Aragona	Salerno
Paolo	Barone	Centro Parkinson Dipartimento Scienze Neurologiche Università Federico II Napoli	Napoli
Marina	Picillo	Centro Parkinson Dipartimento Scienze Neurologiche Università Federico II Napoli	Napoli
Marcello	Moccia	Centro Parkinson Dipartimento Scienze Neurologiche Università Federico II Napoli	Napoli
Stefano	Ruggieri	Neurologia Istituto Mediterraneo Neuromed	Pozzilli
Nicola	Modugno	Neurologia Istituto Mediterraneo Neuromed	Pozzilli
Paolo	Lamberti	Neurologia “Amaducci” Az. Osp. Univ. Policlinico Consorziale	Bari
Claudia	Dell'Aquila	Neurologia “Amaducci” Az. Osp. Univ. Policlinico Consorziale	Bari
Giulio	Cicarelli	Neurologia A.O.R.N. San Giuseppe Moscati	Avellino
Aldo	Quattrone	Clinica Neurologica Università Magna Grecia	Catanzaro
Giuseppe	Nicoletti	Clinica Neurologica Università Magna Grecia	Catanzaro
Antonino	Cannas	Neurologia Policlinico Universitario di Monserrato	Monserrato
Paolo	Solla	Neurologia Policlinico Universitario di Monserrato	Monserrato
Mario	Zàppia	Clinica Neurologica I Policlinico Universitario	Catania
Alessandra	Nicoletti	Clinica Neurologica I Policlinico Universitario	Catania
Letterio	Morgante	Clinica Neurologica Policlinico G. Martino	Messina
Francesca	Morgante	Clinica Neurologica Policlinico G. Martino	Messina
Marco	D'Amelio	Dipartimento di Biomedicina Sperimentale e Neuroscienze Cliniche, Università di Palermo	Palermo
Valeria	Terruso	Neurologia Az. Osp. Univ. Policlinico “P. Giaccone”	Palermo
Roberto	Eleopra	SOC Neurologia Az. Osp. Univ. S.Maria della Misericordia	Udine
Marco	Mucchiut	SOC Neurologia Az. Osp. Univ. S.Maria della Misericordia	Udine
Manuela	Pilleri	U.O. Malattia di Parkinson IRCCS Ospedale San Camillo	Venezia
Roberta	Biundo	U.O. Malattia di Parkinson IRCCS Ospedale San Camillo	Venezia
Stefania	Nassetti	U.O.C. Neurologia Ospedale Bellaria	Bologna
Roberto	Michelucci	U.O.C. Neurologia Ospedale Bellaria	Bologna
